# Prevalence of wasting, overweight and obesity among children under 5 years in 10 cities of Jiangsu Province: a multi-center cross-sectional study

**DOI:** 10.3389/fnut.2026.1679010

**Published:** 2026-01-29

**Authors:** Lihua Li, Hemei Bu, Wen Zheng, Yufei Ni, Aiping Wu, Kan Ye, Xinye Jiang, Guoqiang Yang, Guoqin Liu, Yelin Bao, Li Zhang, Hongxia Qi, Heyun Lv, Rui Qin, Yan Zhao

**Affiliations:** 1 Department of Clinical Nutrition, Lianshui People's Hospital Affiliated to Kangda College of Nanjing Medical University, Huaian, Jiangsu, China; 2 Department of Clinical Nutrition, Jiangsu Province Hospital, The First Affiliated Hospital of Nanjing Medical University, Nanjing, China; 3 Department of Child Health Care, Yancheng Maternity and Child Health Care Institute, Yancheng, China; 4 Department of Child Health Care, Nantong Maternity and Child Health Care Hospital, Nantong, China; 5 Department of Child Health Care, Xinghua Maternity and Child Health Care Hospital, Xinghua, China; 6 Department of Child Health Care, Suzhou Municipal Hospital, Suzhou, China; 7 Department of Child Health Care, Wuxi Maternity and Child Health Care Hospital, Wuxi, China; 8 Department of Child Health Care, Kunshan Maternity and Child Health Care Institute, Kunshan, China; 9 Department of Child Health Care, Dafeng Maternity and Child Health Care Hospital, Dafeng, China; 10 Department of Child Health Care, Drum Tower Maternity and Child Health Care Institute, Nanjing, China; 11 Department of Child Health Care, Huai'an Maternity and Child Health Care Hospital, Huaian, China; 12 Department of Child Health Care, Xuzhou Children's Hospital, Xuzhou, China; 13 Department of Child Health Care, Jiangning Maternity and Child Health Care Institute, Nanjing, China; 14 Department of Child Health Care, Jiangsu Women and Children Health Hospital, Women and Child Branch Hospital of Jiangsu Province Hospital, The First Affiliated Hospital with Nanjing Medical University, Nanjing, China; 15 Department of Nutrition, Affiliated Hospital of Jiangnan University, Wuxi, China; 16 Wuxi School of Medicine, Jiangnan University, Wuxi, China; 17 Institute of Future Food Technology, JITRI, Yixing, China

**Keywords:** children, multi-center study, obesity, overweight, wasting

## Abstract

**Objective:**

To understand the current epidemiological status and influencing factors of wasting, overweight and obesity among children under 5 years old in Jiangsu Province, thereby providing a scientific basis for developing early strategies.

**Methods:**

A multi-center, stratified cluster random sampling approach was employed to investigate birth status, season variation, regional differences and other relevant factors among children under 5 years in Jiangsu Province from April 2014 to March 2015.

**Results:**

A total of 5,289 children were initially enrolled in this study. After applying strict inclusion and exclusion criteria, 4,420 children were included in the statistical analysis (2,303 boys and 2,117 girls). The prevalence of wasting, risk of overweight, overweight and obesity were 1.403, 17.583, 4.594, and 1.426%, respectively. Firstly, the prevalence of wasting was relatively higher in children in rural areas, aged 24–35 months, born via spontaneous delivery, with a birth weight <2,500 g, and investigated in summer and central Jiangsu areas, with prevalence rates of 1.776, 2.009, 1.692, 1.923, 2.070 and 2.760%, respectively. All cities in Jiangsu Province except Nanjing were associated with wasting. Secondly, the prevalence of risk of overweight was higher among boys, infants aged 0–11 months, preterm infant, born with birth weight ≥4,000 g, investigated in winter, with prevalence rates of 19.236, 24.583, 21.304, 23.029 and 24.481%, respectively. And boys, all age groups except 48–59 months, birth weight ≥4,000 g, investigated in spring and winter were factors associated with risk of overweight. Thirdly, the prevalence of overweight was higher among boys, infants aged 0–11 months, birth weight ≥4,000 g, investigated in winter, with prevalence of 5.471, 7.292, 7.884, and 8.605%, respectively. And boys, 0–11 months, birth weight ≥4,000 g, investigated in spring and winter were factors associated with overweight. Finally, the prevalence of overweight among children with boys, 36–47 months, birth weight ≥4,000 g, investigated in winter, and southern of Jiangsu Province were 1.824, 2.039, 2.075, 2.533 and 1.727%, respectively. And boys were factors associated with obesity.\.

**Conclusion:**

The historical prevalence of wasting, overweight and obesity among children under 5 years in Jiangsu Province remain at a low level.

## Introduction

Based on an analysis of 3,663 epidemiological studies spanning over 200 countries involving 220 million children and adolescents, it has been observed that nearly all countries experience the dual burden of pediatric overweight/obesity and wasting (1). With socioeconomic development and improved living standards, the prevalence of childhood overweight and obesity in China has become increasingly significant. The multi-factorial prevention and intervention strategies required for childhood obesity in China remain complex, despite the implementation of the Healthy China 2030 initiative and the adoption of multiple policy by various governmental agencies. Childhood obesity remains an ongoing public health challenge (2). The prevalence rates of overweight and obesity among children younger than 6 years in China are 6.8 and 3.6%, respectively, during 2015–2019 (3). Furthermore, Wang (4) found that the prevalence of wasting was 3.3 and 2.1% among 38,848 children age 0–6 years, based on data from the “2019–2021 Survey and Application of China’s Nutrition and Health System for Children aged 0–18 years in China,” using both China’s standards and WHO standards. These findings suggest that children under 6 years in China are facing a dual burden of malnutrition, characterized by the coexistence of wasting (undernutrition) and overweight/obesity (overnutrition) (5). Malnutrition adversely impacts growth trajectories, development, and overall health outcomes, with impacts that can persist into adulthood, thus representing a significant public health concern (6). Pediatric living with obesity/overweight status are associated with increased risks of type 2 diabetes mellitus (T2DM) (7–9), polycystic ovary syndrome (PCOS) (10, 11), nonalcoholic fatty liver disease (NAFLD) (12) and other chronic conditions (13, 14). Conversely, wasting reflects undernutrition often linked with infections diseases. Wasting, if left untreated, can lead to compromised immune function, impaired neurodevelopment (15), heightened vulnerability to infections, and even death (16).

In addition, research showed that 70% of children with obesity and overweight reside in low-income and middle-income countries (17). Meanwhile, studies also indicated that economic growth was weakly associated with childhood malnutrition and various contributing factors (18). Furthermore, research demonstrated that the prevalence of wasting was generally lower in children under 2 years, based on a cross-sectional study involving 94 mostly low- and middle-income countries (16). Jiangsu Province, located in eastern China, belongs to the Yangtze-river economic zone, is one of the most densely populated and agriculturally productive region in the country with approximately 85 million residents in 2020, exhibiting a gross domestic product (GDP) per capita closely comparable to Beijing and Shanghai (19) and reaching 13.7 trillion yuan ($1.87 trillion) in 2024 (20).

Although significant research has been conducted on childhood obesity globally, data concerning children under 5 years old, particularly in developing countries, remains limited (21, 22). Yu (23) analyzed the prevalence trends of overweight/obesity in Jiangsu Province from 2017 to 2021 based on 5 cross-sectional studies. However, this analysis was confined to 210,168 students aged 6–17 years and lacked data on children under 5 years old. Consequently, Jiangsu Province lacks sufficient sample size and representative data on malnutrition among children under 5 years (24, 25). Therefore, this study employed a multi-center, stratified random cluster sampling methodology to assess the current prevalence and distribution of malnutrition (including wasting, overweight, and obesity) among children under 5 years in Jiangsu Province. The results aim to inform the development of targeted interventions related to physical growth, development, and malnutrition in children under 5 years, both within Jiangsu Province and across China.

## Methods

### Participants and study design

**Figure 1:**
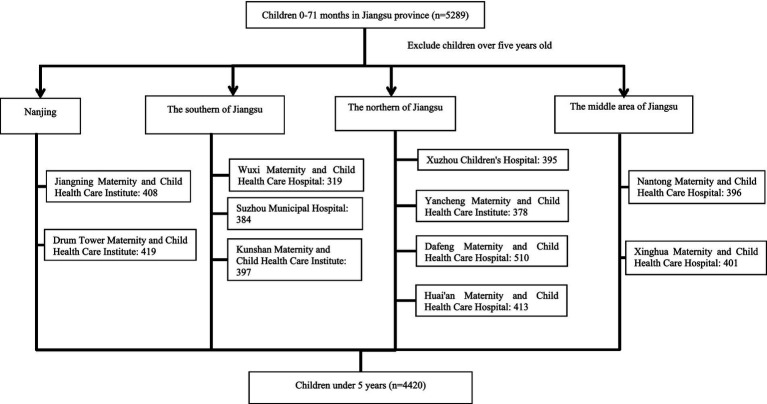
Flowchart of children through the study.

This was a large population-based, cross-sectional multi-center epidemiological study involving children under 5 years in Jiangsu Province, China. Participants were recruited from 11 maternal and child health institutions across 10 cities in Jiangsu Province from April 2014 to March 2015, including Jiangning Maternity and Child Health Care Institute, Drum Tower Maternity and Child Health Care Institute, Kunshan Maternity and Child Health Care Institute, Suzhou Municipal Hospital, Wuxi Maternity and Child Health Care Hospital, Nantong Maternity and Child Health Care Hospital, Xinghua Maternity and Child Health Care Hospital, Yancheng Maternity and Child Health Care Institute, Huai’an Maternity and Child Health Care Institute, Dafeng Maternity and Child Health Care Hospital, and Xuzhou Children’s Hospital. The recruitment framework targeted children attending regular health assessment at these institutions during the study period, with inclusion/exclusion criteria and sampling methods detailed in a previous study (26). For the current study, children under 5 years (4,420) were selected from the original cohort of individuals aged 0–71 months (5,289). Exclusion criteria included age over 5 years (869). Recruitment status at each site is summarized in Figure 1.

In this study, Jiangsu Province was segmented into four regions: Nanjing, the northern region of Jiangsu Province, the central region of Jiangsu Province, and the southern of Jiangsu Province. Three or four pediatric healthcare centers were randomly selected from each region, with geographic distribution detailed in Figure 1. Face-to-face interviews were conducted with parents or caregivers of the children using structured questionnaires, and anthropometric measurements of weight and height/length were obtained following standardized procedures. All protocols adhered to the Declaration of Helsinki by the World Medical Association and complied with the ethical standards of the institutional and national review board responsible for human research. Written informed was obtained from all participants. This study protocol received approval from the Institutional Review Board of the First Affiliated Hospital with Nanjing Medical University (2014-SR-167).

### Assessment of demographic variables

Data on children’s demographic characteristics was collected via structured questionnaire, including gender, birth weight, number of pregnancies, parity, delivery mode, age, gestational age, region of residence, season of survey, and location within Jiangsu Province.

### Anthropometric measurement procedures

According to the measurement methods protocols recommended in the “Technical Specifications for Health Management of Children Aged 0–6 Years” (27) and the “Anthropometric Methods for Population Health Monitoring” (28), length of infants and children under 2 years of age was measured using a recumbent measuring bed with a scale division of 0.1 cm, with readings accurate to 0.1 cm. For children aged 2 years and above, height was measured with a vertical stadiometer with a scale division of 0.1 cm with readings accurate to 0.1 cm. During height/length measurement, children should be bareheaded and barefoot. Children’s weight was measured using a scale with a precision of ≤ 0.01 kg. The child should be stripped of all clothing as much as possible. For children aged 2 years and below, the infant can be wrapped in a blanket of known weight, and the weight readings should be taken when the infant is calm, with an accuracy to 0.01 kg. For children aged 2 years and above, weight should be measured in the early morning on an empty stomach after excretion, using a calibrated scale with a precision of ≤0.1 kg. The child should stand calmly at the center of the scale platform, barefoot and hat-less, and with close-fitting underwear. The scale reading should be recorded, accurately to 0.1 kg.

### Definition of terms

The weight-to-length/height *Z* score (WLZ/WHZ) was calculated according to the World Health Organization Standard (29). In this study, obesity was defined as a ZWHZ >3, overweight as a 2 < WLZ/WHZ ≤ 3, risk of overweight as 1 < WLZ/WHZ ≤ 2 and wasting as a WLZ/WHZ < -2.

### Statistical analysis

Data were double-entered using EpiData software to ensure quality, followed by analysis in SPSS 29.0. Descriptive data are presented as frequencies (n) and percentage (%). Inter group comparisons were conducted using the chi-square test. Multivariate logistic regression analyses were performed to evaluate the distribution of wasting, risk of overweight, overweight, obesity, overweight and obesity among children under 5 years. The Hosmer-Lemeshow test was employed to assess the goodness of fit, Complex survey design adjustments, including specifying clusters as facilities, strata as regions, and sampling probabilities, were incorporated to account for sampling bias. *p* < 0.05 is considered statistically significant.

### Quality control

Conduct standardized training for the medical personnel and investigators involved in the trial, regularly organize personnel exchanged for experience sharing and knowledge updates to ensure procedural consistency. Utilize uniformly designed questionnaires, anthropometric measurement tools, etc., were utilized to guarantee the completeness and accuracy of data collection. Data entry should be prompt, precise, and unambiguous, with any unauthorized alterations strictly prohibited. A data review committee was established to certify the completeness, logic consistency, and outliers within the dataset. Form an independent quality supervision team to conduct regular inspections of all of the trial aspect, including participant follow-up and standardization of data collection.

## Results

This cohort study initially encompassed 5,289 pediatric participants recruited via 11 maternal and child health institutions. Following the exclusion of 869 children aged over 5 years, a final sample of 4,420 children under 5 years was subjected to statistical analysis, including 2,303 boys (52.104%) and 2,117 girls (47.896%) (Table 1). The prevalence rates of wasting, overweight, and obesity among children under 5 years in Jiangsu Province were 1.403, 4.594, and 1.426%, respectively. As shown in Table 1, chi-square analysis revealed no statistically significant differences in the prevalence of wasting, overweight, and obesity concerning household registration, parity, number of births, and whether the birth was full-term (*p* > 0.05). Considering stratification and other potential confounding variables, a supplementary survey analysis was further performed to validate the consistency of the findings. The prevalence (95% confidence intervals) of wasting, risk of overweight, overweight, obesity, overweight were 1.40 (0.66–2.15%), 23.93 (19.31–28.56%), 4.73 (3.18–6.27%), 1.45 (0.80–2.09%) and 6.10 (4.15–8.06%), respectively, indicating that the overall prevalence of wasting, overweight, and obesity remained within the bounds of the corresponding reference ranges or confidence intervals provided in Supplementary Table S1. Regarding the discriminative efficacy as reflected by the Diagnostic Effectiveness Function Teaching (DEFT), the results indicated DEFT value of 4.64 for wasting, suggesting a moderate discriminative capacity. The DEFT for risk of overweight was 13.3, demonstrating a relatively strong ability to identify at-risk individuals. The DEFT for overweight was 5.92, implying moderate performance, while the DEFT for obesity was 3.34, indicating a lower discriminative capacity relative to other outcomes. When overweight and obesity are combined, the DEFT was 7.58, reflecting a moderate-to-strong capacity to identify children with either condition. The strong consistency observed between the data in Table 1 and Supplementary Table S1 underscores the internal reliability of this study, supporting the overall accuracy of the results.

**Table 1:** Current status of wasting, overweight, and obesity in children under 5 years in Jiangsu.

Characters	Group	*N*	Wasting		Risk of Overweight		Overweight		Obesity		*χ^2^*	*P*
			*n*	%	*n*	%	*n*	%	*n*	%		
Age	0–11 months	960	13	1.354	236	24.583	70	7.292	10	1.042	99.595	**<0.001**
	12–23 months	912	14	1.535	162	17.763	38	4.167	6	0.658		
	24–35 months	846	17	2.009	152	17.967	20	2.364	14	1.655		
	36–47 months	883	11	1.246	129	14.609	36	4.077	18	2.039		
	48–59 months	819	7	0.855	98	11.966	39	4.762	15	1.832		
	0–59 months	4,420	62	1.403	777	17.583	203	4.594	63	1.426		
Gender	Boys	2,303	33	1.433	443	19.236	126	5.471	42	1.824	26.599	**<0.001**
	Girls	2,117	29	1.370	344	15.777	77	3.637	21	0.992		
Household registration	Town	3,124	39	1.248	552	17.670	139	4.449	47	1.505	2.836	0.586
	Rural	1,296	23	1.776	225	17.375	64	4.942	16	1.236		
Parity	1 times	3,386	48	1.418	598	17.661	144	4.253	52	1.536	5.068	0.280
	≥2 times	991	13	1.312	167	16.852	57	5.752	11	1.110		
Gestational age	Preterm infant	230	3	1.304	49	21.304	8	3.478	4	1.739	3.559	0.469
	Full-term infant	4,091	59	1.442	695	16.989	187	4.571	55	1.344		
Delivery mode	Spontaneous delivery	1891	32	1.692	310	16.393	66	3.490	27	1.428	13.399	**0.009**
	Cesarean delivery	2,434	30	1.233	437	17.954	133	5.464	34	1.397		
Birth weight	< 2,500 g	156	3	1.923	27	17.308	2	1.282	2	1.282	34.416	**<0.001**
	2,500–4,000 g	3,738	54	1.445	629	16.829	162	4.334	51	1.364		
	≥ 4,000 g	482	3	0.622	111	23.029	38	7.884	10	2.075		
Season of survey	Spring	1,136	9	0.792	196	17.254	55	4.842	15	1.320	87.700	**<0.001**
	Summer	2029	42	2.070	328	16.166	67	3.302	25	1.232		
	Autumn	581	7	1.205	88	15.146	23	3.959	6	1.003		
	Winter	674	4	0.593	165	24.481	58	8.605	17	2.533		
Location in Jiangsu Province	Nanjing	827	3	0.363	141	17.050	29	3.507	11	1.330	38.551	**<0.001**
	The southern of Jiangsu	1,100	20	1.818	161	14.636	52	4.727	19	1.727		
	Central region of Jiangsu	797	22	2.760	143	17.942	31	3.890	10	1.255		
	The northern of Jiangsu	1,696	17	1.002	332	19.575	91	5.366	23	1.356		

The bolded values mean *p* < 0.05, signifying statistical significance.

Statistically significant differences were observed in terms of gender, age, mode of delivery, birth weight, survey season, and region. The prevalence of wasting among children with spontaneous delivery (1.692%) was higher than those with cesarean delivery (1.233%), whereas the prevalence of overweight (5.464%) among children with cesarean delivery were higher than those of children with spontaneous delivery. Seasonally, prevalence of wasting was highest in summer (2.070%), while the prevalence of overweight (8.605%) and obesity (2.533%) were highest in winter. Children with birth weight ≥ 4,000 g had the lowest prevalence of wasting (0.622%) and the highest prevalence of overweight (7.884%) and highest prevalence of obesity (2.075%).

**Figure 2:**
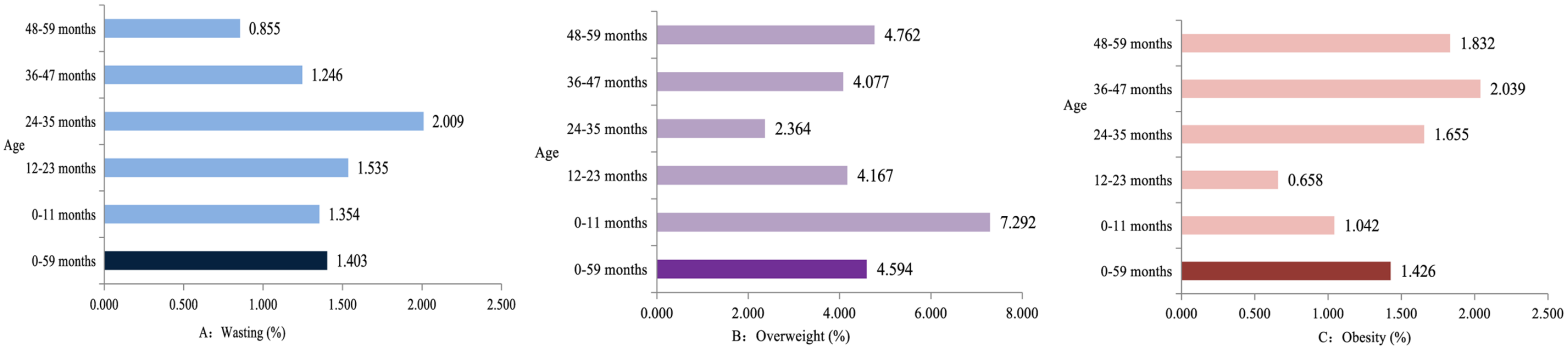
The prevalence of wasting, overweight, and obesity among children under 5 years in Jiangsu. (**A–C**, %).

As illustrated in Figure 2, the highest prevalence of wasting (2.009%) was observed in children aged 24–35 months, while the lowest prevalence of wasting (0.855%) occurred in children aged 48–59 months (Figure 2A). The highest prevalence of overweight (7.292%) was found in children aged 0–11 months, and the lowest prevalence of obesity (2.364%) was observed in children aged 24–35 months (Figure 2B). The highest prevalence of obesity (2.039%) was noted in children aged 36–47 months, and the lowest prevalence of obesity (0.658%) was recorded in children aged 12–23 months (Figure 2C).

**Figure 3:**
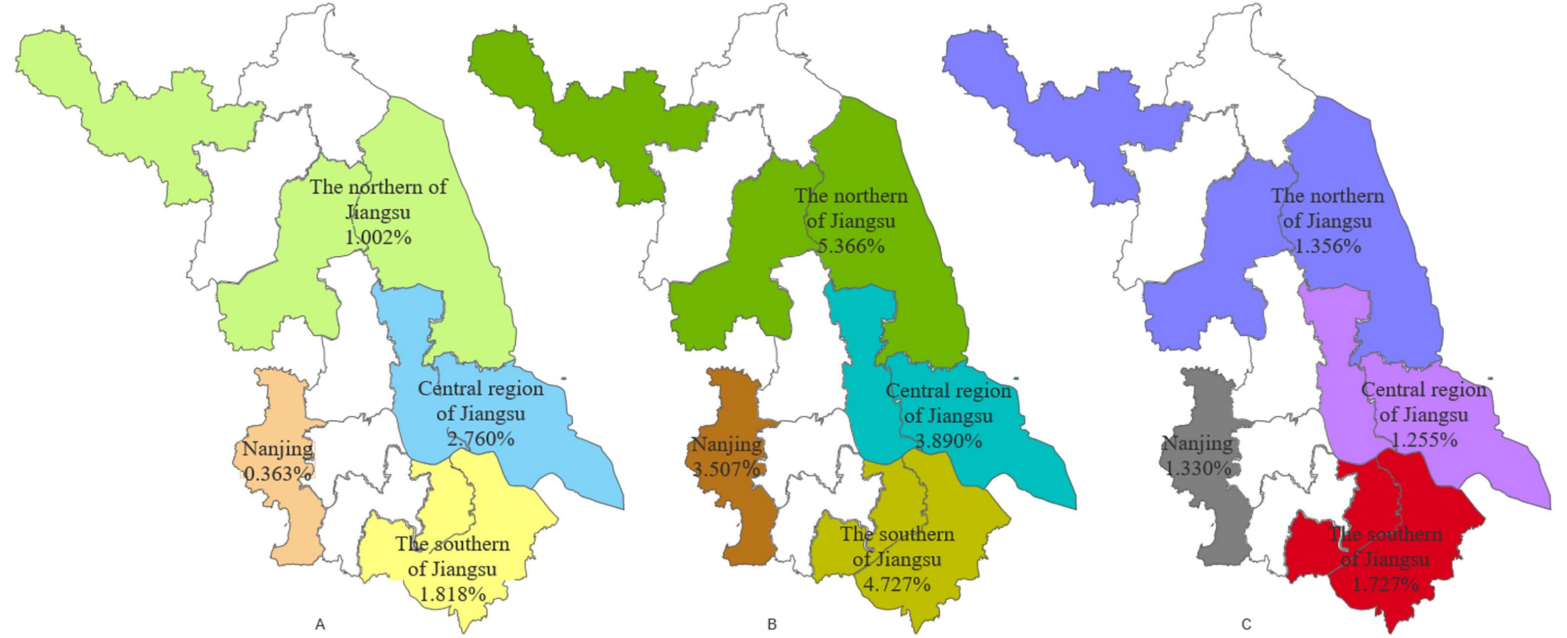
Regional distribution of wasting, overweight, and obesity among children under 5 years in Jiangsu province **(A–C)**.

As shown in Figure 3, Nanjing exhibited the lowest prevalence of wasting (0.363%, Figure 3A) and overweight (3.507%, Figure 3B), while southern Jiangsu Province displayed the lowest prevalence of overweight (14.71%, Figure 3B) and obesity (1.727%, Figure 3C). Central Jiangsu recorded the highest rate of wasting (2.760%, Figure 3A), and northern Jiangsu had the highest rates of overweight (5.366%, Figure 2B).

**Figure 4:**
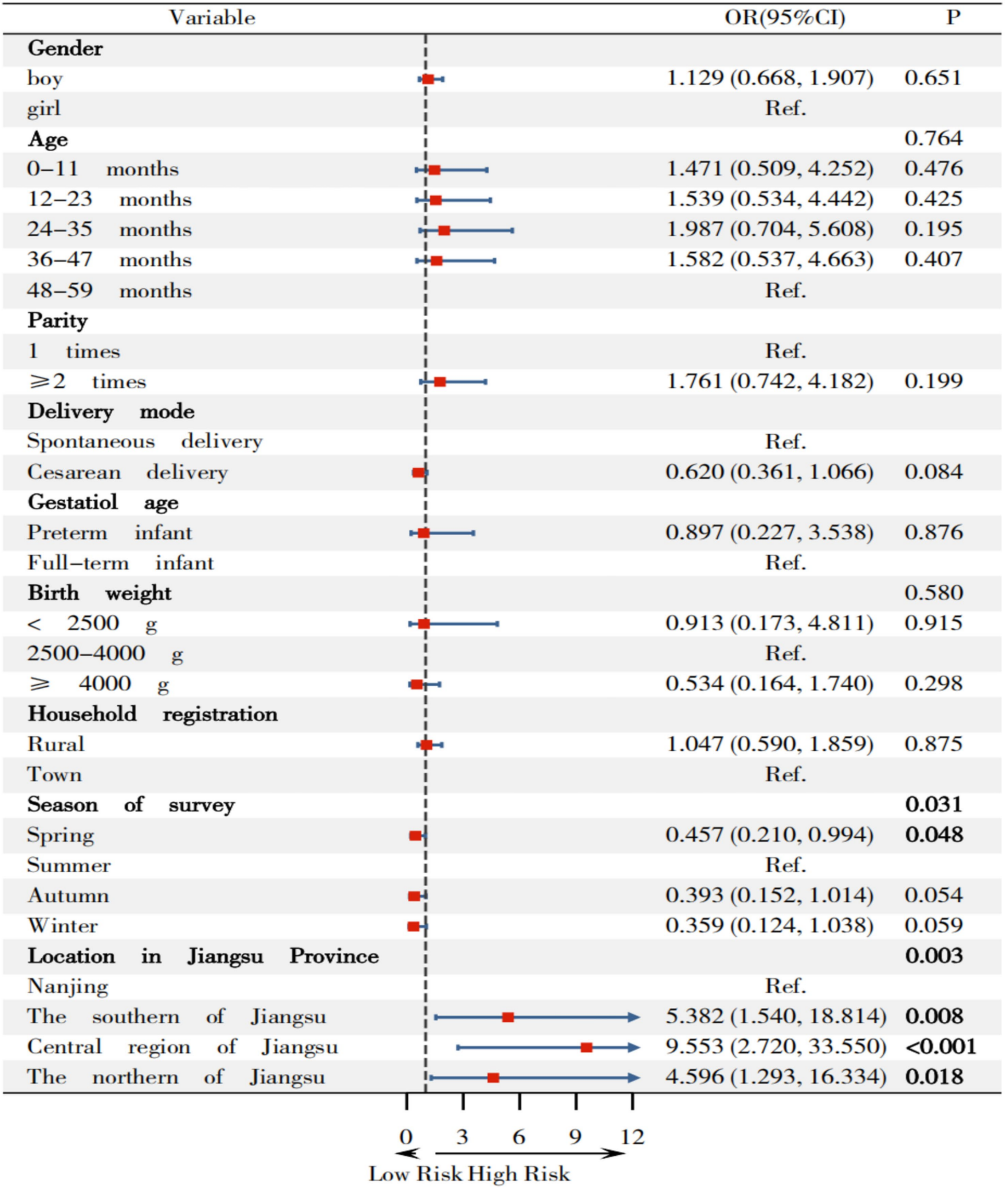
Relationship between variables on wasting among children under 5 years in Jiangsu.

According to Figure 4, compared to surveys in summer, the odds ratios (OR) and 95% CI for wasting among children surveyed in spring was 0.457(0.210, 0.994). Furthermore, compared to Nanjing, OR (95%CI) for wasting in southern Jiangsu, central Jiangsu, and northern Jiangsu were 5.382(1.540, 18.814), 9.553(2.720, 33.550), and 4.59 6(1.293, 16.334), respectively.

**Figure 5:**
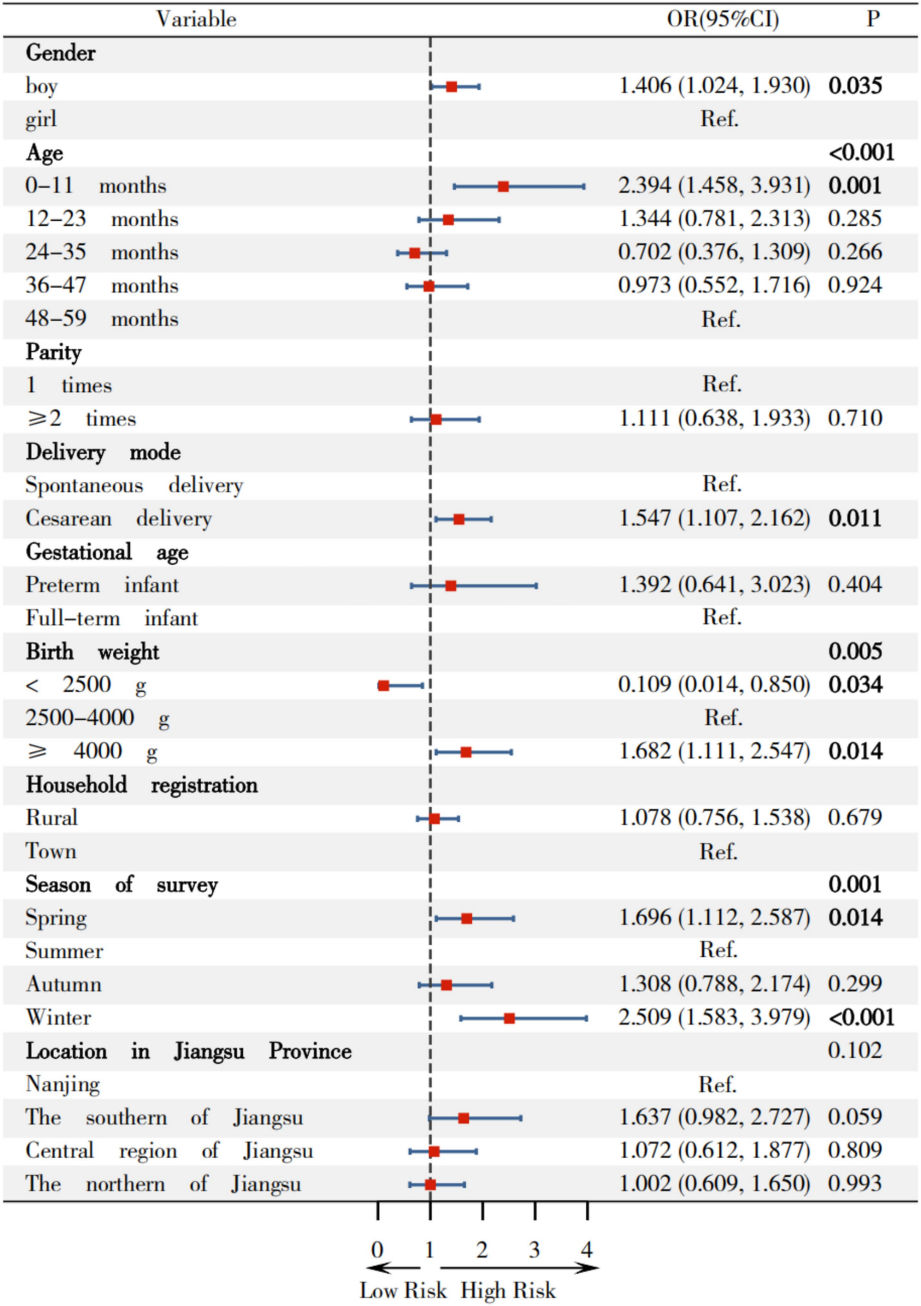
Relationship between variables on overweight among children under 5 years in Jiangsu.

In Figures 5, compared to girl, boys demonstrated a higher risk of overweight, with OR(95%CI) of 1.406(1.024, 1.930). Children aged 0–11 months showed an OR(95%CI) of 2.394(1.458, 3.931) for overweight, compared to children with 48–59 months. Children with a birth weight of ≥4,000 g had an OR(95%CI) of 1.682(1.111, 2.547) for overweight relative to children with normal birth weight. Conversely, children with birth weights <2,500 g exhibited a significantly lower OR of 0.109 (95% CI: 0.014–0.850). In comparison to surveys conducted in summer, OR(95%CI) for overweight in children surveyed in spring and winter were 1.696(1.112, 2.587) and 2.509(1.583, 3.979), respectively.

**Figure 6:**
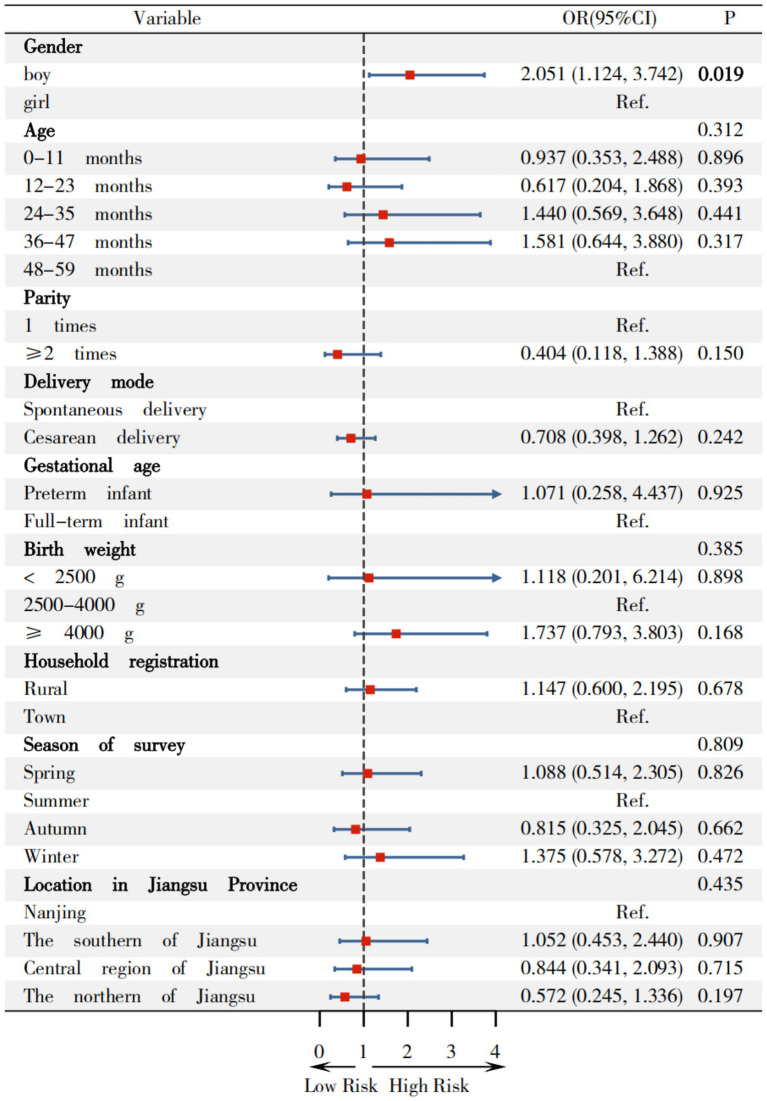
Relationship between variables on obesity among children under 5 years in Jiangsu.

In Figure 6, boys had higher odds of obesity compared to girl, with an OR(95%CI) of 2.051 (1.124, 3.742). The multivariate logistic regression analysis indicates that no other covariates show statistically significant associations with obesity status.

**Figure 7:**
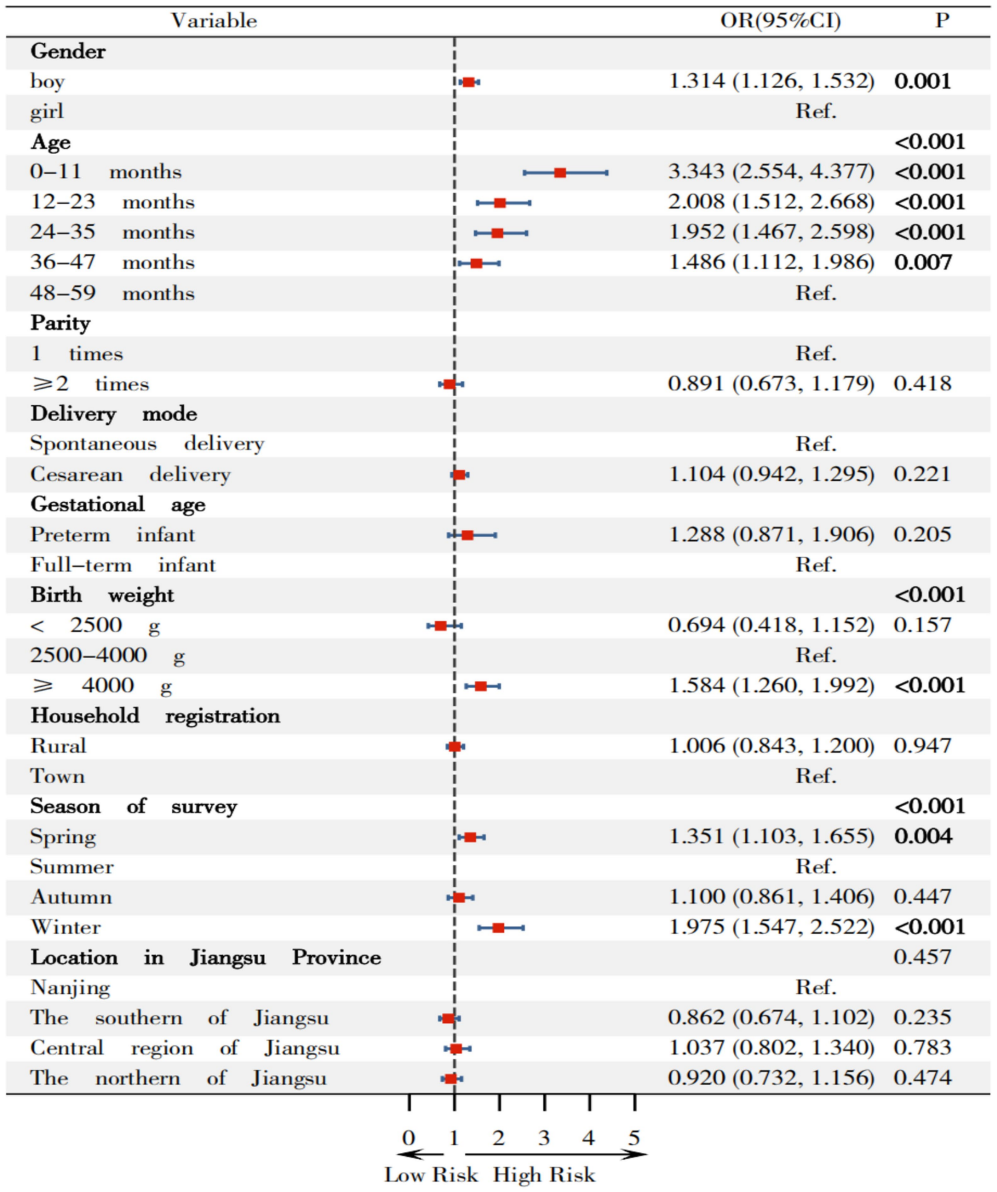
Relationship between variables on risk of overweight among children under 5 years in Jiangsu.

In Figure 7, children with birth weights <2,500 g exhibited a significantly lower OR of 0.109 (95% CI: 0.014–0.850). Children aged 0–11 months, 12–23 months, 24–35 months, 36–47 months displayed increased odds of overweight compared to those aged 48–59 months, with OR(95%CI)s of 3.343 (2.554–4.377), 2.008 (1.512–2.668), 1.952 (1.467–2.598), and 1.486 (1.112–1.986) for respective age groups. Children with birth weights ≥4,000 g showed elevated odds with OR(95%CI) of 1.584 (1.260–1.992) relative to normal-weight children. The OR(95%CI)s for overweight during spring and winter compared to summer were 1.351 (1.103–1.655) and 1.975 (1.547–2.522), respectively.

**Figure 8:**
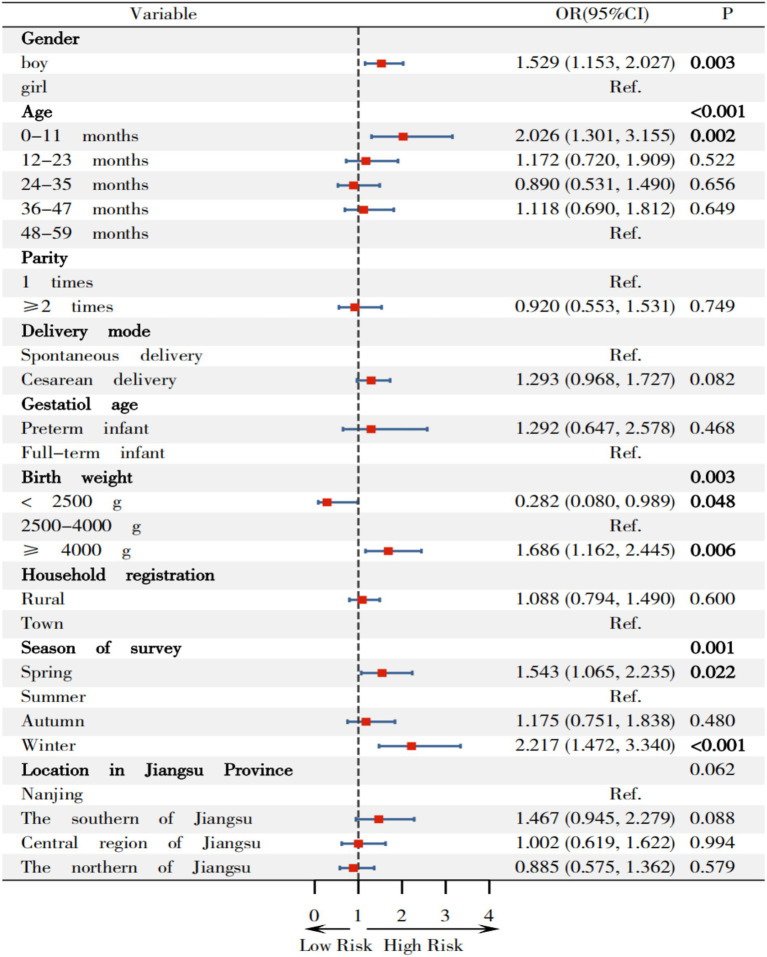
Relationship between variables on overweight and obesity among children under 5 years in Jiangsu.

Figure 8 revealed that boys have higher odds of combined overweight and obesity compared to girls, with an OR(95%CI) of 1.529 (1.153, 2.027). Children aged 0–11 months also showed increased odds compared to children with 48–59 months, with an OR(95%CI) of 2.026 (1.301, 3.155). Children with birth weights ≥4,000 g had an OR(05%CI) of 1.686 (1.162, 2.445) relative to normal-weight children, while those with weights <2,500 g had significantly lower odds with an OR(95%CI) of 0.282 (0.080, 0.989), comparing children with normal birth weight. Compared to summer, the OR(95%CI)s for overweight and obesity in children surveyed in spring and winter were 1.543 (1.065, 2.235) and 2.217 (1.472, 3.340), respectively.

## Discussion

Although the data-set employed in this study was collected between 2014 and 2015, Jiangsu Province has historically consistently lacked comprehensive, large-scale epidemiological surveillance data on pediatric malnutrition-related morbidity, including wasting, overweight, and obesity among children aged 0 to 5 years. Consequently, epidemiological data from over a decade ago remain critically relevant, serving as a fundamental resource for elucidating temporal trends and phenotypic patterns. Even with accelerated socioeconomic development in Jiangsu Province over the past decade, the historical prevalence rates of childhood wasting, overweight, and obesity from 2014 continued to function as key epidemiological benchmarks for current public health intervention strategies. Such longitudinal data underpin ongoing public health decision-making and policy development by informing the design, adjustment, and implementation of targeted, evidence-based nutritional interventions. The continued relevance of this data underscores the vital importance of epidemiological surveillance in informing effective, sustainable strategies for childhood nutritional health within the region.

## Current status of wasting, overweight and obesity in Jiangsu

Firstly, WHO data (30) indicates that the global wasting rate among children under 5 years was 6.8% in 2022. Md Moyazzem Hossain (31) showed that the prevalence of wasting in children was 8% based on a study of 8,334 children under 5 years in Bangladesh. A study by Li et al. (32) involving 299,353 children aged 1–5 years from 35 countries found a wasting rate of 12.9% among data for LMICs from DHSs conducted between 2007 and 2018. Furthermore, Wang (4) found the prevalence of wasting was 3.3% in 38,848 children under 6 years from 14 provinces, autonomous regions or municipalities in China. Zhang (33) reported that the wasting rate was 1.2% from 110,491 children under 7 years from 9 cities located in the northern, central and southern region of China in 2016. Additionally, the prevalence of wasting among was 1.08% among 82,278 children under 5 years in Zhang’s study. This study, conducted with 4,420 children, shows that the prevalence of wasting among children under 5 years in Jiangsu Province is 1.403%, indicating that historical prevalence of wasting in Jiangsu Province is relatively low both globally and within China.

Secondly, WHO data (30) indicates that the global overweight rate among children under 5 years was 5.59% in 2022. The study conducted by Pan (3) indicated that the prevalence of overweight among Chinese children younger than 6 years was 6.8% based on Chinese criteria. Wang (34) analyzed data from the Institute for Health Metrics and Evaluation (IHME) and found that the prevalence of overweight among children under 5 years of age increased from 17.9% (95% CI: 14.9 to 21.3) in 2000 to 22.1% (95% CI: 14.9 to 30.9) in 2019. Yu (24) investigated 32,861 Chinese children aged under 6 years in 2013, revealing that the prevalence of overweight ranged from 3.3 to 16.1%, with the highest being 16.1% in Chongqing (16.1%). Furthermore, the prevalence of overweight among children under 6 years in Jiangsu (1,218 children) was 7.7%. This study indicates that the prevalence of overweight among children under five in Jiangsu Province is 4.594%, suggesting that the historical prevalence of overweight among children under 5 years in Jiangsu Province is relatively low in China.

Thirdly, a meta-analysis (35) based on 2,033 studies from 154 different countries or regions involving 45,890,555 individuals performed on general population between January 2000 and March 2023, showing the overall prevalence of obesity in children and adolescents was 8.5% (95% CI 8.2–8.8). Furthermore, 246 articles in this meta-analysis involving 7,839,060 children indicated that the global prevalence of obesity among children under 5 years is 8.46%. Hong’s study (36) revealed that the prevalence rate of obesity among Chinese children under 6 years is 3.6%. Yu (24) found that the prevalence rate of obesity ranged from 0.6 to 9.7% after surveying 32,861 children under 6 years in 2013. In Yu’s study, the prevalence rate of obesity was 3.3% in Jiangsu Province. Li et al. (37) found that the prevalence of obesity were 2.75, 2.63 and 2.40% among 51,460 preschoolers aged 1–6 years from 2021 to 2023 in Beijing, China. This study showed that the historical prevalence of obesity among children under 5 years in Jiangsu Province is 1.426%, indicating that the prevalence of childhood obesity in Jiangsu Province is relatively low on both globally and within China.

## Effects of basic variables on wasting among children in Jiangsu

Firstly, this study indicated that the prevalence of wasting (2.070%) is the highest among children surveyed in summer, and compared to this group, children surveyed in spring exhibit a lower wasting rate (0.792%) and a protective risk [OR(95%CI) = 0.457 (0.210, 0.994)], which align with previous studies. Tusting (38) indicated that high temperatures can lead to decreased appetite in children based on a study involving 656,107 children under 5 years in sub-Saharan Africa. There was a significant association between high temperatures and stunted growth in children (such as wasting and underweight), which is consistent with the findings in this study. Sandler (39) also found that a maximum monthly temperature of 38 °C in the preceding growing season was associated with a 25% prevalence of wasting among 48,086 Nigerian children and 28,421 Kenyan children. That is the higher temperature corresponds to a 10-percentage point increase in higher prevalence of wasting. Therefore, temperature levels play a significant role in determining wasting prevalence.

Secondly, this study indicates that children in southern Jiangsu, central Jiangsu, and northern Jiangsu regions have a higher risk of wasting, compared to Nanjing, with OR (95%CI)s of 5.382 (1.540, 18.814), 9.553 (2.720, 33.550), and 4.596 (1.293, 16.334), respectively. Li et al. (32) showed that poor economic conditions was one of the factors associated with child wasting, based on a cross-sectional study of 299,353 children aged 12–59 months in 35 low- and middle-income countries. Zhang (33) found that region was one of the factors associated with malnutrition in the multivariate logistic analysis among 110,491 children under 7 years from 9 cities in China, which is consistent with the findings of this study.

## Effects of basic variables on overweight and obesity among children in Jiangsu

Firstly, compared to girls, the prevalence of overweight (5.471%), risk of overweight (19.236%), obesity (1.824%), and combined overweight and obesity (6.020%) in boys, as well as their OR(95%CI) values [1.406 (1.024, 1.930), 1.314 (1.126, 1.532), 2.051 (1.124, 3.742) and 1.529 (1.153, 2.027)] were higher than those in girls, which is largely consistent with existing findings. Yu (40) analyzed data from 32,862 children in the “2010–2013 China National Nutrition and Health Status Survey” showing that the prevalence of overweight and obesity among boys under 6 years (9.4 and 3.6% respectively) were higher than those among girls (7.2 and 2.5% respectively). Liu (5) found that overweight/obesity was associated with males in a cross-sectional study conducted among 7,664 Chinese children aged 2–6 years in Hunan Province. Moore’s research (41) indicated that there were gender differences in growth and neurocognitive development during infancy and early childhood, with boys having greater demands for growth and maintenance. Chen’s research (42) of 56,738 children aged 2–7 in Xiamen showed that physiological differences between boys and girls may lead to boys being more prone to fat accumulation. Liang et al. (43) found that boys exhibited higher prevalence of overweight and obesity than girls in a sampling of 193,997 Chinese children aged 3–18 years from 11 provinces. Regrettably, Liang et al. did not recruit 0–3 years children and investigated the prevalence of overweight and obesity and associated factors. Vasanthakumar’s study (44) also demonstrated that gender plays a significant role in childhood overweight and obesity. Therefore, the influence of gender on overweight and obesity in children under 5 years involves multiple aspects, including biology, socio-cultural, and familial factors.

Secondly, the highest prevalence of overweight and risk of overweight were observed in children aged 0-11 months (7.292 and 24.583%, respectively), while the highest prevalence of obesity was observed in children aged 36–47 months (2.039%). Conversely, the lowest prevalence of overweight and risk of overweight was observed in children aged 24–35 months (2.364%) and 48–59 months (11.966%), respectively, which slightly differ from existing research. Compared to children aged 48–59 months, the OR (95%CI) values for overweight, combined overweight and obesity among children aged 0–11 months were 2.394 (1.458, 3.931) and 2.206 (1.301, 3.155). Zhang (33) operated a cross-sectional survey among 110,491 children under 7 years in 9 cities located in the northern, central, and southern region of China in 2016, found that the highest prevalence of overweight (3.0%) and obesity (1.3%) was observed in children aged 48–59 months, while the lowest prevalence of overweight (2.1%) and obesity (0.3%) were observed in children aged 24–35 months and 0–23 months among 82,279 children under 5 years. Yu (40) analyzed data from 32,862 children under 6 years in the “2010–2013 China National Nutrition and Health Status Survey” revealing that the lowest prevalence of overweight among 27,820 children under 5 years occurred in those aged 0–11 months (12.0%) and the lowest prevalence of obesity was in those aged 48–59 months (3.9%). This study consistent with the findings of Yu. The discrepancy between the highest and lowest age groups for overweight among children under 5 years underscores the need for ongoing research in this field.

Thirdly, this study revealed that children with a birth weight ≥4,000 g demonstrated the highest prevalence of overweight (7.884%), risk of overweight (23.029%) and obesity (2.075%). Compared to children with a normal birth weight, the OR(95%CI)s for overweight, risk of overweight, and combined overweight and obesity in children with a birth weight ≥4,000 g were 1.682 (1.111, 2.547), 1.584 (1.260, 1.992) and 1.686 (1.162, 2.445), respectively, suggesting that infants with a high birth weight are at increased risk of developing overweight and obesity later in life, consistent with existing research literature. Conversely, birth weight <2,500 g was associated with protective effects against overweight with OR(95%CI) value of 0.109 (0.014, 0.850). Zong (45) identified birth weight ≥4,000 g was one of the 12 factors associated with of childhood obesity in a population-based matched case–control study of 63,292 preschool children aged 3–7 years across 9 cities in China. Pan (46) reported that macrosomic infants had 1.90-fold increased risk of childhood obesity compared to infants with normal birth weight in a cohort study of 1,767 infants (including 714 macrosomic infants) in western China,. Multiple studies have confirmed that birth weight ≥ 4,000 g is an independent factor associated with overweight and obesity in children (47). Jiang’s meta-analysis (48) also identified high birth weight as one of the 33 factors associated with childhood obesity from incorporating 419 studies. Liang et al. (43) similarly found that birth weight >4,000 g was the factors associated with overweight and obesity among children under 5 years.

Finally, this study reveals that the prevalence of overweight, risk of overweight, and combined overweight and obesity in children were the highest during the winter surveys, with increased odds [OR(95%CI): 2.509 (1.583, 3.979), 1.975 (1.547, 2.522) and 2.217 (1.472, 3.340), respectively] when compared with surveyed in summer. Similar trends were observed in spring [OR(95%CI): 1.696 (1.112, 2.587), 1.351 (1.103, 1.655) and 1.543 (1.065, 2.235), respectively]. These findings are consistent with existing research, indicating that seasonal variations influence children’s appetite and metabolism through various mechanisms (49–51). Wang (49) observed higher prevalence of overweight (18.03%) and obesity (6.89%) in a cross-sectional study of 305 preschool children aged 3–6 in the Greater Khingan Mountains, suggesting cold temperature significantly impact nutritional status of children. Shahar (50) confirmed that winter is typically associated with increased red meat intake, which reflects seasonal effects on dietary pattern, assessed via semi-quantitative food frequency questionnaire (including 96 categories) among 94 individuals. Waswa (51) demonstrated that the dietary diversity scores and food variety of children were significantly higher in November than in July/August among 426 mother–child (children aged 6–23 months) pairs in western Kenya. Additionally, Weaver (52) found that movement behavior (sedentary activity, physical activity, and sleep) are associated with the risk of obesity in children in a systematic review and meta-analysis of 47 studies involving 27,093 children. These behaviors may vary throughout the year due to changes in weather, daylight, and climate, with children being least active in their movement behaviors during the summer and winter.

This study indicates that the prevalence of wasting, overweight and obesity among children under 5 years in Jiangsu Province is relatively low. The samples were collected from 11 medical institutions across the province using a multi-center stratified random design, which ensures a certain level of representativeness. The findings provide a basis for government departments to formulate targeted prevention and intervention strategies.

The aim of this study was to investigate the current status and distribution of wasting, overweight, and obesity among children under 5 years in Jiangsu Province. Given the multi-center design and regional stratification, the findings may provide insights into pediatric nutritional status within similar healthcare settings in Jiangsu Province, although generalizability to broader populations should be considered with caution due to the convenience sampling framework and site-specific participation rates. It should be noted that this study has certain limitations. Firstly, this study focused on prevalence of wasting, overweight, and obesity among children under five in Jiangsu Province, analyzing the impact of pertinent demographic determinants. The surveillance predominantly elucidated patterns of pediatric epidemiological patterns of wasting, overweight and obesity, considering variables such as temporal trends, geographic stratification, and demographic profiles. Due to spatial limitations of this paper, comprehensive data on perinatal, postnatal, and childhood lifestyles factors (including dietary, nutrition supplements, sleep and outdoor activity) collected during the survey were not extensively addressed within this publication. Secondly, anthropometric status in this study was assessed using WLZ/WHZ. Future studies should further examine associations between body composition (e.g., lean mass and fat mass) and the distribution of fat mass (visceral vs. subcutaneous). However, these limitations highlight directions for our future research, which is expected to provide additional guidance and evidence for the prevention of wasting, overweight, and obesity in children.

## Data Availability

The raw data supporting the conclusions of this article will be made available by the authors, without undue reservation.
