# Supplementary material for: Prevalence of wasting, overweight and obesity among children under 5 years in 10 cities of Jiangsu Province: a multi-center cross-sectional study
Source: Front Nutr. 2026 Jan 29;13:1679010. doi: 10.3389/fnut.2026.1679010 (PMC12894040; doi:10.3389/fnut.2026.1679010)
Supplement: Supplementary file 1 [file Table_1.DOCX]

Table 1 Calculate prevalence rates and DEFT

| Outcome | Prevalence (%) | 95% CI | DEFT |
| --- | --- | --- | --- |
| Wasting | 1.40 | 0.66% - 2.15% | 4.64 |
| Risk of overweight | 23.93 | 19.31% - 28.56% | 13.3 |
| Overweight | 4.73 | 3.18% - 6.27% | 5.92 |
| Obesity | 1.45 | 0.80% - 2.09% | 3.34 |
| Overweight and obesity | 6.10 | 4.15% - 8.06% | 7.58 |
